# Improved Survival, Vascular Differentiation and Wound Healing Potential of Stem Cells Co-Cultured with Endothelial Cells

**DOI:** 10.1371/journal.pone.0016114

**Published:** 2011-01-24

**Authors:** Dora C. S. Pedroso, Ana Tellechea, Liane Moura, Isabel Fidalgo-Carvalho, João Duarte, Eugénia Carvalho, Lino Ferreira

**Affiliations:** 1 Centre for Neuroscience and Cell Biology, University of Coimbra, Coimbra, Portugal; 2 Biocant, Centro de Inovação em Biotecnologia, Cantanhede, Portugal; 3 Instituto de Biologia Molecular e Celular, Porto, Portugal; 4 Crioestaminal, Cantanhede, Portugal; University of Milan-Bicocca, Italy

## Abstract

In this study, we developed a methodology to improve the survival, vascular differentiation and regenerative potential of umbilical cord blood (UCB)-derived hematopoietic stem cells (CD34^+^ cells), by co-culturing the stem cells in a 3D fibrin gel with CD34^+^-derived endothelial cells (ECs). ECs differentiated from CD34^+^ cells appear to have superior angiogenic properties to fully differentiated ECs, such as human umbilical vein endothelial cells (HUVECs). Our results indicate that the pro-survival effect of CD34^+^-derived ECs on CD34^+^ cells is mediated, at least in part, by bioactive factors released from ECs. This effect likely involves the secretion of novel cytokines, including interleukin-17 (IL-17) and interleukin-10 (IL-10), and the activation of the ERK 1/2 pathway in CD34^+^ cells. We also show that the endothelial differentiation of CD34^+^ cells in co-culture with CD34^+^-derived ECs is mediated by a combination of soluble and insoluble factors. The regenerative potential of this co-culture system was demonstrated in a chronic wound diabetic animal model. The co-transplantation of CD34^+^ cells with CD34^+^-derived ECs improved the wound healing relatively to controls, by decreasing the inflammatory reaction and increasing the neovascularization of the wound.

## Introduction

It is estimated that 15% of the diabetic patients have non-healing foot ulcerations [1], [2]. In recent years, there have been efforts to develop new advanced methodologies to heal chronic wounds, including the use of topic growth factors or cell-based therapies [3]. Some of these therapies have been approved by the Food and Drug Administration (FDA); however, more effective ways for the treatment of chronic wounds are needed, especially in diabetic patients [1], [4].

Recent data show that healthy adult stem/progenitor cells improve the healing of diabetic chronic wounds [5]. It has been shown that peripheral blood-derived CD34^+^ cells, but not CD34^-^ cells, can accelerate the vascularization and healing of diabetic wounds [5]. However, the vasculogenic potential of adult blood-derived cells appears to be reduced by diabetes [5], [6]. Recent studies tried to overcome this issue by using fetal [7] or adult mesenchymal stem cells [8], yet, the isolation of stem cells from fetal aorta poses several problems for future clinical application, while mesenchymal stem cells isolated from diabetic patients might have impaired properties due to ageing and disease.

Human umbilical cord blood (UCB) can be a potential source of healthy endothelial progenitor cells for the healing of chronic wounds in diabetic patients. These cells are obtained non-invasively, can be stored for more than 15 years without loosing biological properties, and they have low immunogenicity, which makes them an interesting candidate for allogeneic transplantation [9], [10]. Improvement in wound healing has been reported recently in two human non-diabetic patients who received topically UCB-derived CD34^+^ cells in a fibrin gel [11], although the underlying regenerative mechanism has not be determined. Despite this potential, human umbilical cord blood stem cells have not been used for wound healing in diabetic patients, whose healing process is impaired or even inexistent.

In the present study, we investigated the use of UCB-derived CD34^+^ cells to promote the healing of diabetic wounds when administered topically in a fibrin gel. To enhance the therapeutic effect of CD34^+^ cells, they were co-cultured with endothelial cells (ECs) derived from CD34^+^ cells. *In vitro* data show that CD34^+^-derived ECs co-cultured with CD34^+^ cells improve cell survival and contribute to the differentiation of CD34^+^ cells into ECs. We further show that the co-culture system, but not CD34^+^ cells or CD34^+^-derived ECs alone, can improve the healing kinetics in a diabetic animal model. The regenerative effect is mediated by both anti-inflammatory and pro-angiogenic processes. We anticipate that this co-culture approach might be used in other contexts to enhance the efficacy of stem cells.

## Materials and Methods

An expanded Materials and Methods section is provided in the online data supplement (**Material and Methods S1)**.

### Isolation of CD34^+^ cells from UCB

All human umbilical cord blood samples were collected from donors, who signed an informed consent form, in compliance with Portuguese legislation. The collection was approved by the ethical committee of Hospital Infante D. Pedro. The samples were stored in sterile bags containing 35 mL of citrate-phosphate-dextrose anticoagulant solution. CD34^+^ cells were isolated from mononuclear cells, obtained from UCB samples after Ficoll (Histopaque-1077 Hybri Max; Sigma-Aldrich, St. Louis, USA) density gradient separation. CD34^+^ cells were positively selected (2 times) using the mini-MACS immunomagnetic separation system (Miltenyi Biotec, Bergisch Gladbach, Germany, http://www.miltenyibiotec.com), according to the manufacturer's recommendations. CD34^+^ cells were immediately used for cell encapsulation studies or *in vivo* experiments without further treatment.

### Differentiation of CD34^+^ cells into ECs

Isolated CD34^+^ cells were transferred onto 1% (w/v) gelatin-coated 24-well plates (2×10^5^ cells/well) and incubated in endothelial growth medium (EGM-2; Lonza, Gaithersburg, MD, USA) with 20% (v/v) fetal bovine serum (FBS; Invitrogen, Carlsbad, USA) and 50 ng/mL vascular endothelial growth factor (VEGF_165_; PrepoTech Inc., Rocky Hill, USA), at 5% CO_2_, 37°C. After 5 days and then every other day, half of the volume of the medium was replaced with fresh one. At the end of the differentiation assay, expression of EC markers was evaluated by fluorescence-activated cell sorting (FACS) and immunofluorescence staining. The functionality of the cells was evaluated by incubating the cells with acetylated low-density lipoprotein (DiI-Ac-LDL; Biomedical Technologies, Stroughton, USA).

### Cell encapsulation in fibrin gels

The fibrin gel preparation procedure can be found in the online data supplement (**Material and Methods S1)**. CD34^+^ cells (between 0.5×10^5^ and 3×10^5^), CD34^+^ cells (1×10^5^) in co-culture with CD34^+^-derived ECs (0.35×10^5^), CD34^+^-derived ECs (0.35×10^5^) or human umbilical vein endothelial cells (HUVECs) (Lonza) (0.35×10^5^) were encapsulated in fibrin gels (50 µL). Cells were centrifuged and ressuspended in the fibrin gel precursor solution, in 1 mL sterile syringes with cut tips. Polymerization was initiated at 37°C and allowed to proceed over 30 min. After polymerization, the cell constructs were removed from the syringe and placed in 24-well plates containing specific medium (see below for details), for up to 10 days. Viability and metabolic activity of encapsulated cells were evaluated according to methodologies described in the online data supplement (**Material and Methods S1)**.

### Cell characterization

Detailed protocols for FACS, immunostaining and quantitative reverse transcription-polymerase chain reaction (qRT-PCR) analysis are presented in the online supplement **Material and Methods S1** and primer sequences on **Table S1**.

### Cytokine secretion analyses

Cell culture supernatants and protein lysates were evaluated for the presence of cytokines using a Bio-Plex Pro Human Cytokine 17-Plex Panel Assay or a Bio-Plex Pro Mouse Cytokine 8-Plex Assay, respectively (both from Bio-Rad, Hercules, CA, USA), according to manufacturer's instructions, and further described in the online supplement.

### Analysis of total and phosphorylated Akt and ERK protein levels

Activation of Akt and extracellular signal-regulated kinase (ERK) in CD34^+^ cells and CD34^+^-derived ECs was promoted by starving the cells for 19 h in serum-free M199 medium (with Earle's Salts and L-glutamine; Sigma-Aldrich) and then treating them for 10 min, by replacing the medium by fresh M199 or cell-conditioned M199. Following treatment, proteins were isolated from the cells, and the levels of Akt and ERK phosphorylation determined using Bio-Plex kits from Bio-Rad, according to manufacturer's instructions.

### CD34^+^ cell adhesion to different substrates

A 24-well plate was coated with several substrates, including fibrin gel (500 µL per well), collagen type I gel (500 µL per well, from a solution of 2.5 mg/mL rat tail collagen I; BD Biosciences Pharmingen, San Jose, CA, USA), or fibronectin (Roche Applied Science, Mannheim, Germany) [50 µg/mL, in Phosphate Buffer Saline (PBS; Sigma-Aldrich)]. CD34^+^ cells labeled with 5(6)-carboxyfluorescein diacetate N-succinimidyl ester (CFSE) (Sigma-Aldrich) and suspended in EGM-2 medium (1×10^5^/mL) were seeded on the coated or uncoated (polystyrene) wells and incubated for 3 h at 37°C. After this time, the culture medium was removed and the cells attached to the wells washed twice with PBS. Attached cells were then counted after photographing six random microscope fields (×200 magnification) for each replicate.

### CD34^+^ cell adhesion and differentiation on top of fibrin gels

Fibrin gel precursor solution (500 µL per well) was added to a 24-well plate and allowed to polymerize. For the adhesion assay, CFSE-labeled CD34^+^ cells (1×10^5^, unless otherwise stated) were seeded on top of a fibrin gel and cultured in EGM-2 medium, either alone or in co-culture with CD34^+^-derived ECs (0.35×10^5^), or in CD34^+^-derived EC conditioned medium. The conditioned medium was obtained by culturing CD34^+^-derived ECs in EGM-2 medium for 48 h, at a density of 0.75×10^5^ cells per mL (24-well plate), removing the medium and sterilizing it by filtration. The number of adherent CD34^+^ cells was determined in a phase-contrast microscope or in a fluorescence microscope after 7 days of culture, following the removal of the non-adherent cells, by washing with PBS. The differentiation potential of CD34^+^ cells (1×10^5^) on top of fibrin gels was assessed in similar conditions. After 7 days, the ability of the CD34^+^ cells to uptake DiI-Ac-LDL was determined by fluorescence microscopy and compared to the initial level of uptake of DiI-Ac-LDL by CD34^+^ cells immediately following their isolation.

### *In vivo* experiments

Animal protocols (see online data for further details) were approved by the Ethics Committee of the Faculty of Medicine of the University of Coimbra (ID 2008/001). Diabetes mellitus was induced by intraperitoneal injection of streptozotocin (Sigma-Aldrich) in male C57BL/6 mice (10-12 week-old), purchased from Charles River (Wilmington, MA, USA). Only animals with glucose levels greater than 300 mg/dL were used in this study.

Six to eight weeks following diabetes induction, the animals were anesthetized, the dorsolumbar skin shaved and disinfected and two 6 mm-diameter dorsal full-thickness excisional wounds were created with a sterile biopsy punch in each animal. The wounds were then covered with 25 µL PBS, 25 µL fibrin gel alone, or 25 µL fibrin gel precursor solution containing 1×10^5^ human umbilical cord blood CD34^+^ cells (unless otherwise stated), either alone or in combination with 0.35×10^5^ CD34^+^-derived ECs. All cells had been previously labeled with CFSE. After surgery, the animals were maintained in individual cages with food and water *ad libitum* and in a temperature and humidity-controlled environment. The rate of wound closure was determined over ten days. At the end of the study, animals were sacrificed by cervical dislocation and 10 mm-diameter skin biopsies analyzed by RT-PCR, immunofluorescence or Bio-Plex Pro Mouse Cytokine 8-Plex Assay.

### Statistical analysis

For analysis involving three or more groups, ANOVA was used, followed by a Student-Newman-Keuls post hoc test. For analysis of two groups, a paired *t*-test was used. Statistical analysis was performed using GraphPad Prism software (San Diego, CA, USA, http://www.graphpad.com/). Results were considered significant when *P*≤0.05.

## Results

### Differentiation of UCB-derived CD34^+^ cells into ECs

**Figure 1:**
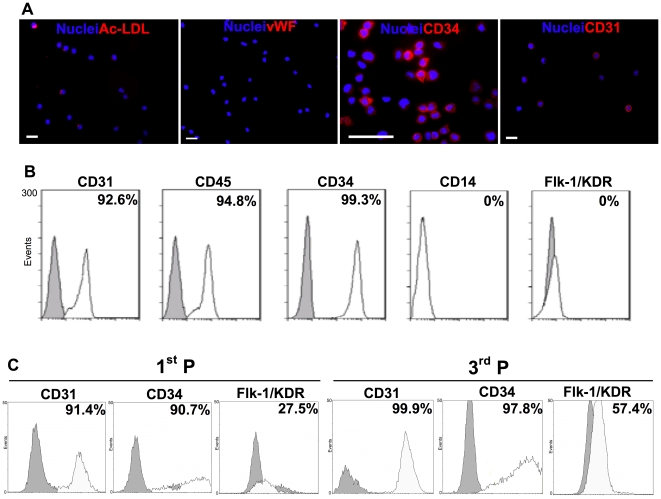
Protein expression in undifferentiated CD34^+^ cells and their endothelial progenies. A) Immunofluorescence results for undifferentiated CD34^+^ cells. Bar corresponds to 20 µm. B, C) FACS analysis of (B) undifferentiated CD34^+^ cells and (C) CD34^+^-derived ECs at passage 1 (1^st^ P) and 3 (3^rd^ P). Percentages of positive cells were calculated based on the isotype controls (grey plot) and are shown in the histogram plots.

**Figure 2:**
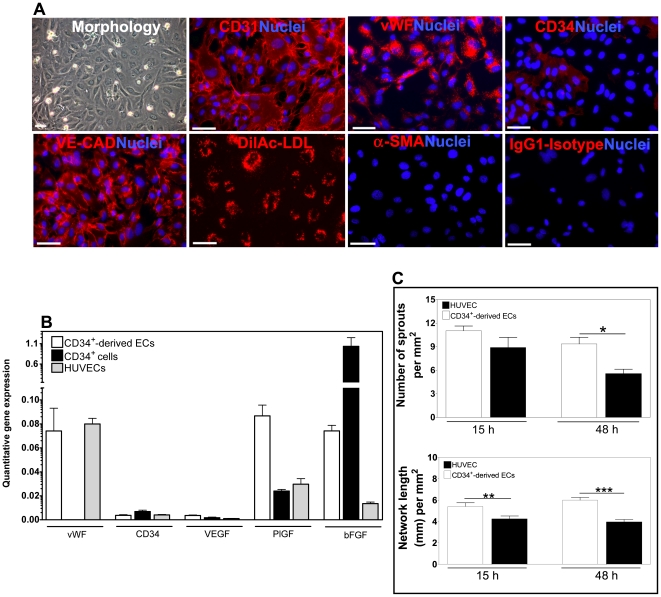
Characterization of CD34^+^-derived ECs. A) Immunofluorescence analysis. Bar corresponds to 40 µm. B) Quantitative RT-PCR analysis. Results are average ± SD, *n* = 4. C) Quantification of cord length and number of sprouts at 15 h and 48 h. Counts were performed using a magnification of ×100. Results are average ± SD, *n* = 3. *, **, *** denote statistical significance (*P*<0.05, *P*<0.01, *P*<0.001, respectively).

Results obtained by immunocytochemistry show that undifferentiated CD34^+^ cells express high levels of CD34 (100%) and CD31 markers (85.4±17.0%, *n* = 8), incorporate moderate levels of Ac-LDL (12.8±10.4%, *n* = 13) and show no expression of the definitive endothelial marker vWF (**Fig. 1A**). FACS analyses confirm their high expression of CD34 and CD31 markers, high expression of CD45, a typical marker of hematopoietic cells, and no expression of Flk-1/KDR and CD14 (a marker mainly expressed by macrophages) (**Fig. 1B**). Similarly, no vWF expression and high CD34 expression were observed at gene level (**Fig. 2B**). Therefore, the UCB-derived CD34^+^ population used in this work is CD34^+^CD45^+^CD31^+^KDR^−^vWF^−^CD14^−^.

To differentiate CD34^+^ cells into ECs, we cultured CD34^+^ cells (100,000 cells per cm^2^) in gelatin-coated dishes in EGM-2 medium with 20% (v/v) of FBS and 50 ng/mL of VEGF_165_. Typically after 15–20 days of culture, CD34^+^ cells attached to the culture dish with a cobblestone-like morphology and expressed high levels of EC markers, including CD31, CD34 and Flk-1/KDR over time (**Fig. 1C**). In addition, differentiated CD34^+^ cells stained positively for CD31 and VE-cadherin at cell-cell adherent junctions, produced vWF and were able to incorporate Ac-LDL (**Fig. 2A**), typical markers found in ECs (**Fig. S1**). In line with the EC phenotype, these cells do not express α-SMA, a smooth muscle cell marker (**Fig. 2A**).

Quantitative RT-PCR (qRT-PCR) analyses showed that CD34^+^-derived ECs express the EC markers CD34 and vWF and produce angiogenic growth factors including VEGF, PlGF and bFGF (**Fig. 2B**). The expression of PlGF growth factor is higher on CD34^+^-derived ECs than on CD34^+^ cells, while the opposite was observed for bFGF. In addition, qRT-PCR results show that the expression of angiogenic growth factors is higher on CD34^+^-derived ECs than on HUVECs.

The ability of CD34^+^-derived ECs to form cord-like structures was also assessed by culturing these cells in the basement membrane Matrigel. Similarly to HUVECs, CD34^+^-derived ECs were able to spontaneously reorganize into cord-like structures when maintained in culture for 15 h (**Fig. S2**). Yet, the angiogenesis potential of CD34^+^-derived ECs is higher than that of HUVECs, as confirmed by the higher number of sprouts and network length (**Fig. 2C**).

### Fibrin gels as three-dimensional scaffolds for CD34^+^ and CD34^+^-derived ECs

**Figure 3:**
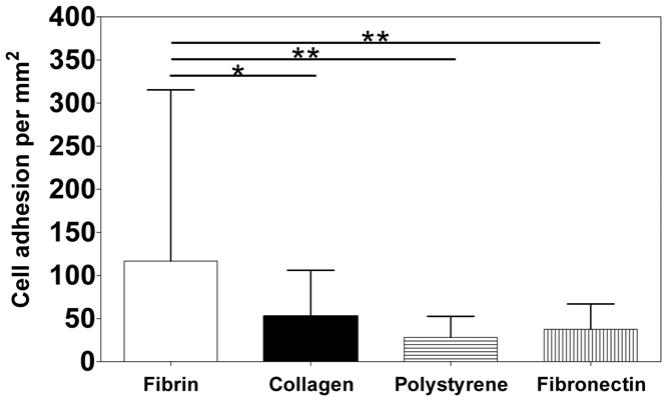
Adhesion of CD34^+^ cells to different substrates. CD34^+^ cells were seeded on 24-well plates coated with fibrin gel, type I collagen gel (2.5 mg/mL) and fibronectin (50 µg/mL), and incubated for 3 h at 37°C. Polystyrene culture wells were used as control. After that time, the cells were washed and the attached ones were counted in six random microscope fields (×200 magnification) for each replicate. Results are average ± SD, *n* = 6. * and ** denote statistical significance (*P*<0.05 and *P*<0.01, respectively).

Fibrin has been shown to provide a permissive environment for endothelial cell adhesion, migration and three-dimensional organization [12], [13]. However, it is unclear whether fibrin gels might be an effective substrate for CD34^+^ cell adhesion. For this purpose, CD34^+^ cells were plated on 24-well plates coated with fibrin gels or different substrates and cell adhesion evaluated by microscopy, by counting the cells attached after 3 h. CD34^+^ cells demonstrated greater initial adhesion to fibrin gels than to polystyrene dishes, collagen and fibronectin (**Fig. 3**). The number of CD34^+^ cells adherent to fibrin was 2.0- (*P*<0.05), 4.0- (*P*<0.01) and 3.0-fold (*P*<0.01) greater than to collagen, polystyrene and fibronectin, respectively.

Next, we used fibrin gels to encapsulate CD34^+^ cells. Initially, we studied the effect of cell number to gel volume for optimal cell survival. Cell viability did not change significantly after 10 days for the different cell densities tested (**Fig. S3**). Therefore, 100,000 CD34^+^ cells per 50 µL of fibrin gel were used in subsequent tests. Fibrin gels resist the degradation initiated by the metalloprotease enzymes released by CD34^+^ cells, or CD34^+^-derived ECs (35,000 cells), or the co-culture of CD34^+^ cells with CD34^+^-derived ECs (100,000: 35,000 cells, respectively) for 10 days (**Fig. S4**). Furthermore, cell traction does not reduce significantly the gel diameter over time in all the experimental groups (**Fig. S4**).

### Cell survival is improved by co-culturing CD34^+^ cells with CD34^+^-derived ECs

**Figure 4:**
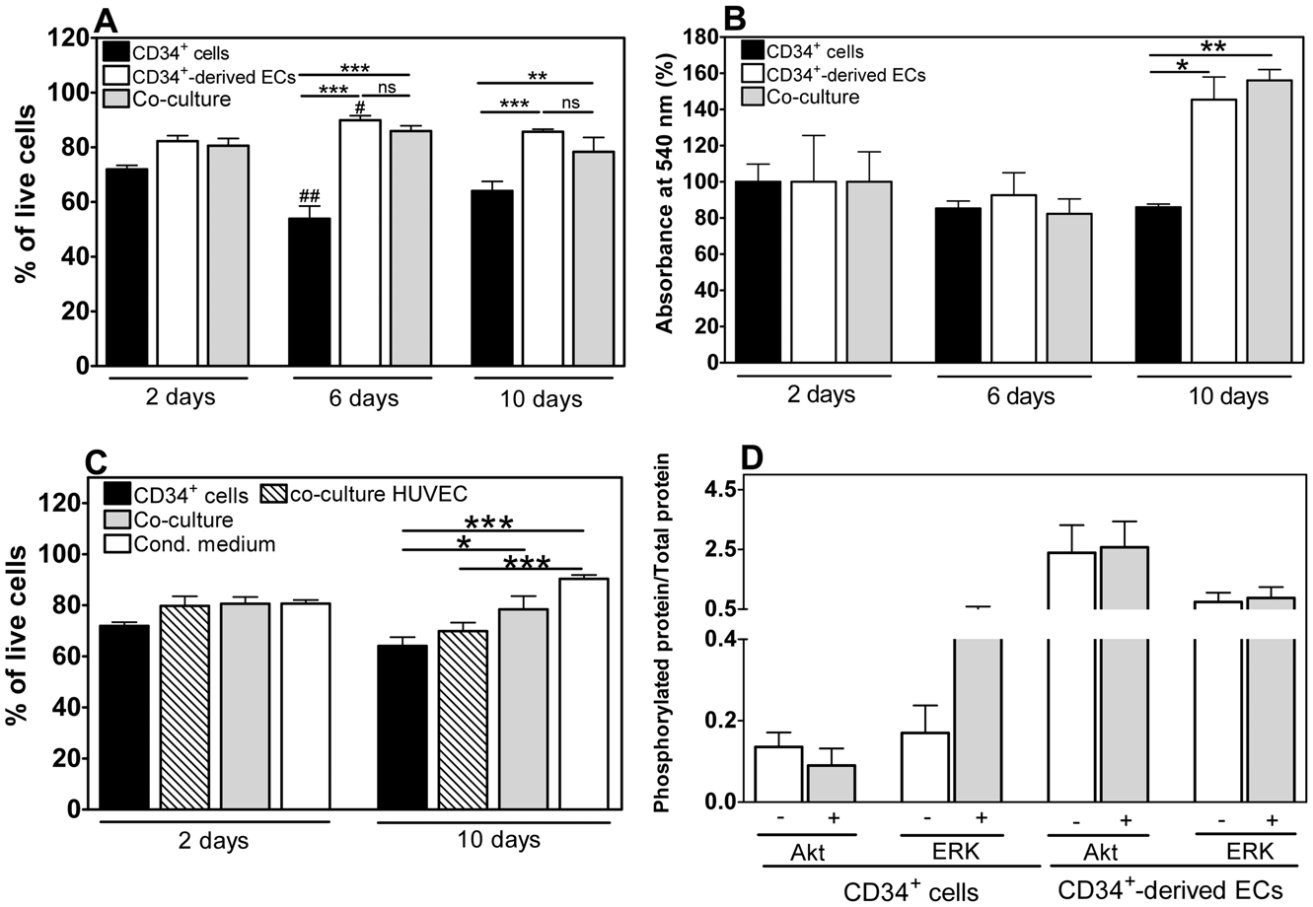
Viability of encapsulated cells. A) Viability of encapsulated cells, as assessed by a LIVE/DEAD assay. Results are average ± SD, *n* = 3. B) Mitochondrial metabolic activity of encapsulated cells. Results are average ± SD, *n* = 6. Each absorbance at 540 nm was normalized by day 2 absorbance. C) Viability of encapsulated cells, as quantified by a LIVE/DEAD assay. Results are average ± SD, *n* = 3. In all figures, * denote statistical significance within time group: * *P*<0.05, **P<0.01, *** *P*<0.001. # denotes statistical significance between time groups comparing the respective control/treatment groups: # *P*<0.05, ## *P*<0.01. D) Phosphorylated Akt/total Akt and phosphorylated ERK/total ERK ratios assessed by a Bio-Plex phosphoprotein detection assay. Results are average ± SD, *n* = 3.

CD34^+^ cells encapsulated in fibrin gels have a viability of 60-70% at day 2 and day 10, statistically lower (*n* = 6, *P*<0.001) than the one observed for CD34^+^-derived ECs (**Fig. 4A**). An MTT assay was used to determine the metabolic activity of cells encapsulated in the gels (**Fig. 4B**). The metabolic activity of CD34^+^ cells at day 10 was approximately 90% of the initial activity (day 2), suggesting that cells were in a non-proliferative state.

Quantitative LIVE/DEAD and MTT analyses indicate a significant increase in cell viability and proliferation for CD34^+^ cells co-cultured with CD34^+^-derived ECs (*n* = 6, *P*<0.01 or *P*<0.001) (**Figs. 4A and 4B**). This effect was observed for days 6 and 10, although more pronounced at day 10, in terms of metabolic activity. In contrast, HUVECs did not affect the viability of CD34^+^ cells (*n* = 6, *P*>0.05) (**Fig. 4C**), indicating that the pro-survival effect is specifically mediated by CD34^+^-derived ECs. Importantly, addition of CD34^+^-derived EC-conditioned medium to cell constructs formed by CD34^+^ cells resulted in a significant increase in cell viability (*n* = 6, *P*<0.001) (**Fig. 4C**). These results suggest that the pro-survival effect of CD34^+^-derived ECs on CD34^+^ cells is mediated, at least in part, by bioactive factors released from the ECs.

Phosphoinositide-3 kinase (PI3K)/Akt and mitogen activated protein kinase (MAPK)/extracellular signal regulated kinase 1/2 (ERK 1/2) pathways regulate several cellular processes, including cell survival [14], [15]. Therefore, we analyzed the activation of Akt and ERK pathways on CD34^+^ cells or CD34^+^-derived ECs cultured in serum free-medium for 19 h and then treated with EC or CD34^+^ cell-conditioned medium, respectively. The ratios between phosphorylated and total proteins were determined by a Multiplex assay (**Fig. 4D**). Interestingly, the ERK but not the Akt pathway is activated in CD34^+^ cells by EC-conditioned medium. Therefore, the pro-survival effect of EC-conditioned medium in CD34^+^ cells seems to be mediated by the activation of the ERK pathway. On the other hand, no significant activation has been observed for ERK and Akt pathways in ECs by CD34^+^-conditioned medium, as compared to the control.

**Figure 5:**
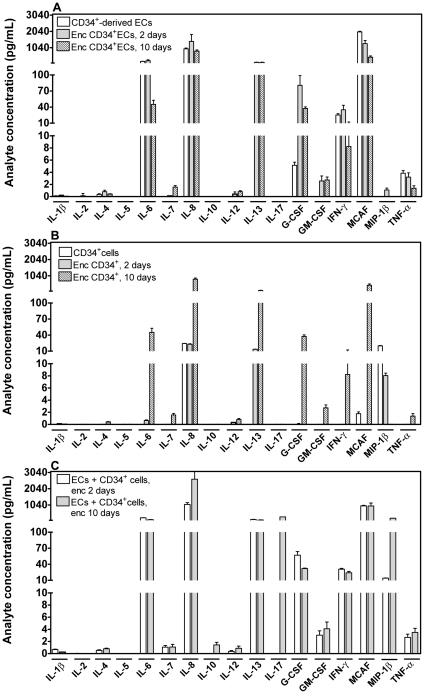
Multiplex cytokine analysis. Analysis of cytokines secreted by (A) CD34^+^-derived ECs grown on tissue culture polystyrene or encapsulated in fibrin gels for 2 or 10 days, (B) CD34^+^ cells grown on tissue culture polystyrene or encapsulated in fibrin gels for 2 or 10 days and (C) co-culture of CD34^+^ and CD34^+^-derived ECs encapsulated in fibrin gels for 2 or 10 days. Cell media were collected after being in contact with the cells for 2 days. Results are average ± SD, *n* = 3.

To understand the crosstalk between both cells, we analysed the cytokines secreted by CD34^+^ cells, CD34^+^-derived ECs, or both cells encapsulated in fibrin gels, using a cytokine bead array (**Fig. 5**). CD34^+^-derived ECs encapsulated in fibrin gels secrete high levels (>100 pg/mL) of IL-6, IL-8, IL-13 and monocyte chemotactic activating factor (MCAF) (**Fig. 5A**). On the other hand, CD34^+^ cells encapsulated in fibrin gels secrete high levels (>100 pg/mL) of IL-8, IL-13 and MCAF (**Fig. 5B**). Importantly, when both cells are encapsulated together in fibrin gels, they secrete high levels of the previous cytokines (IL-6, IL-8, IL-13 and MCAF) and two additional cytokines, IL-10 and IL-17 (**Fig. 5C**). These results confirm the existence of a crosstalk between CD34^+^-derived ECs and CD34^+^ cells.

### CD34^+^ cell adhesion to fibrin gels is enhanced in the presence of CD34^+^-derived ECs or EC-conditioned medium

**Figure 6:**
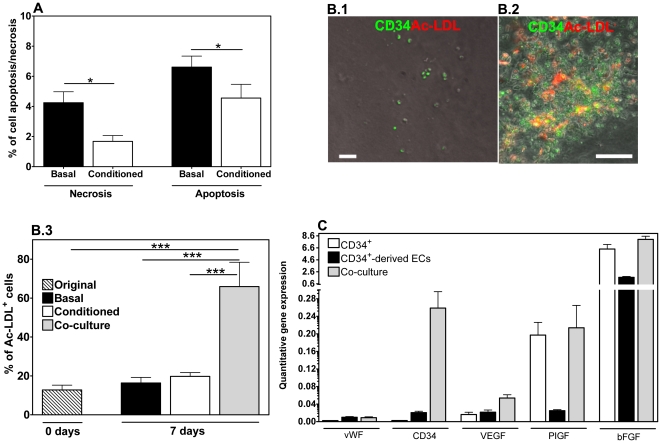
Viability and differentiation of CD34^+^ cells cultured on top of fibrin gels and gene expression analysis of encapsulated cells. A) Apoptosis and necrosis of CD34^+^ cells cultured for 7 days on top of fibrin gels, in EGM-2 medium or EGM-2 medium conditioned by CD34^+^-derived ECs. Results are average ± SD, *n* = 3 (magnification of ×100). B) CD34^+^ cell differentiation on top of fibrin gels for 7 days in the absence (B.1) or in the presence (B.2) of CD34^+^-derived ECs. B.3 shows the percentage of cells able to uptake Ac-LDL. Results are average ± SD, *n* = 18. C) Quantitative RT-PCR analysis of CD34^+^, CD34^+^-derived ECs, or a co-culture of both cells, encapsulated in fibrin gels for 10 days. Results are average ± SD, *n* = 4. In all figures, * denote statistical significance: * *P*<0.05, **P<0.01, *** *P*<0.001.

To investigate the effect of the co-culture system in supporting CD34^+^ cell adhesion, isolated CD34^+^ cells (100,000 cells/well) were cultured on top of fibrin gels (i) in the presence of CD34^+^-derived ECs (35,000 cells/well), (ii) in the absence of ECs, or (iii) in the absence of ECs but in the presence of EC-conditioned medium. To ascertain that any difference in adhesion that might be noticed in the co-culture condition would be due to the presence of ECs and not to cell number, 135,000 CD34^+^ cells were also cultured alone. After 7 days in culture, the non-adherent cells were removed by washing and the adherent cells were counted by phase-contrast microscopy. 5.5±10.4% (*n* = 17), 5.8±3.3% (*n* = 23), 22.4±20.9% (*n* = 15) and 44.2±33.2% (*n* = 18) of CD34^+^ cells were attached to the gel when cultured in the absence of ECs (initial 100,000 and 135,000 CD34^+^ cells), in the presence of ECs, or in the absence of ECs but in the presence of EC-conditioned medium, respectively (**Fig. S5**). CD34^+^ cell adhesion was low when cells were cultured alone, regardless of initial cell number. The differences in CD34^+^ cell recovery cannot be explained by differences in cell apoptosis/necrosis, since only a small fraction (below 10%) of the cells are affected by those (**Fig. 6A**). However, cell apoptosis and necrosis are significantly discouraged (*P*<0.05) when the cells are cultured in EC-conditioned medium rather than basal medium.

### CD34^+^ cell differentiation into ECs is enhanced in the presence of CD34^+^-derived ECs

Next, we assessed the potential of the co-culture system to support CD34^+^ cell differentiation into ECs. For that purpose, CD34^+^ cells (100,000 cells/well) were cultured on top of fibrin gels (i) in the presence of CD34^+^-derived ECs (35,000 cells/well), (ii) in the absence of ECs, or (iii) in the absence of ECs but in the presence of EC-conditioned medium. After 7 days in culture, the ability of the cells to take up Ac-LDL, an endothelial marker, was evaluated by fluorescence microscopy (**Fig. 6B**). Uptake of DiI-labeled Ac-LDL increased from 12% on isolated CD34^+^ cells to 70% after differentiation in the presence of CD34^+^-derived ECs (*P*<0.001). A non-statistically significant increase was observed for Ac-LDL uptake on CD34^+^ cells cultured on fibrin without cells (basal medium, 7 days), compared to original CD34^+^ cells (time zero). Therefore, the results indicate that CD34^+^ cell differentiation into ECs is significantly improved in the presence of CD34^+^-derived ECs. Because no statistical increase in Ac-LDL uptake was observed for CD34^+^ cells cultured in EC-conditioned medium, the endothelial differentiation of CD34^+^ cells is mediated by additional factors to the ones secreted by the CD34^+^-derived ECs.

We also evaluated by qRT-PCR the differentiation of CD34^+^ cells in co-culture with ECs and encapsulated in fibrin gels for 10 days (**Fig. 6C**). The expression of EC markers (vWF and CD34) was similar or higher than for ECs or CD34^+^ cells encapsulated in fibrin gels, indicating that most of the cells in the gels express endothelial markers.

### Co-transplantation of CD34^+^ cells with CD34^+^-derived ECs improves wound healing in diabetic mice

**Figure 7:**
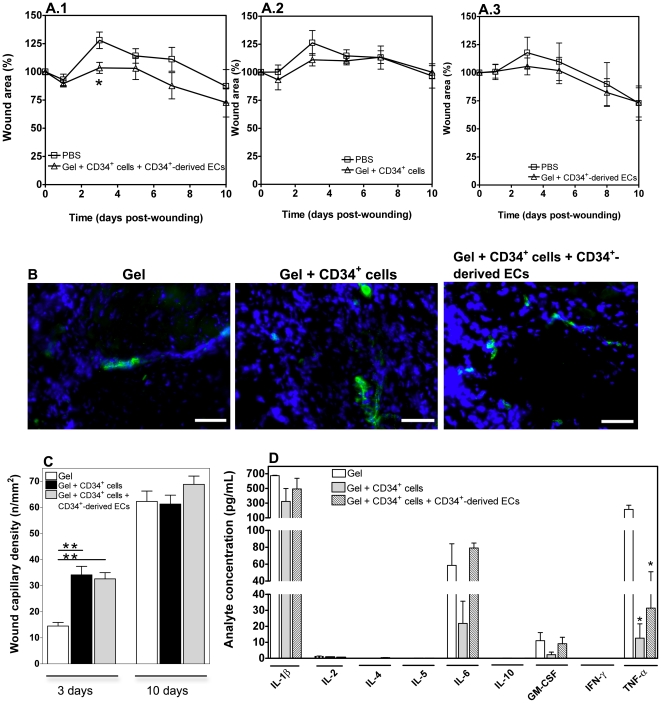
Regenerative effect of encapsulated CD34^+^ cells and CD34^+^-derived ECs on diabetic wounds. A) Wound closure (relatively to initial wound area) in diabetic mice treated by topical application of 1×10^5^ CD34^+^ cells and 0.35×10^5^ CD34^+^-derived ECs (A.1), CD34^+^ cells (A.2) or CD34^+^-derived ECs (A.3), encapsulated in fibrin gels. Control wounds received a saline solution (PBS) only. Results are average ± SEM, *n* = 6. * denotes statistical significance (*P*<0.05). B) Representative images of vWF immunostaining of wounds at day 3. Scale bar represents 50 µm. C) Quantification of wound capillary density (number of capillaries per mm^2^), based on the vWF immunostaining results, for 3 and 10 day-old wounds in the different experimental groups. Results are average ± SEM, *n* = 3. ** denotes statistical significance (*P*<0.001). D) Cytokine expression on mouse wound samples excised 3 days post-wounding, as determined by a Bio-Plex mouse cytokine assay. Wounds had been treated by topical application of CD34^+^ cells or CD34^+^ cells plus CD34^+^-derived ECs encapsulated in fibrin gels. Control wounds received fibrin gel alone. Results are average ± SD, *n* = 3.

The therapeutic effect of our tissue engineering approach was further evaluated in a chronic wound animal model. Wounds were created at the anterior and posterior dorsal regions of streptozotocin-induced diabetic mice. One of the wounds was covered with fibrin gel alone or PBS to serve as internal control, whereas the other wound was covered with gel containing 1×10^5^ CD34^+^ cells, or 0.35×10^5^ CD34^+^-derived ECs, or 1×10^5^ CD34^+^ cells with 0.35×10^5^ CD34^+^-derived ECs. At day 3 post-wounding, the wound area was statistically lower in wounds treated with the gel containing a co-culture of CD34^+^ cells with ECs compared to wounds treated with PBS (*P*<0.05, *n* = 6), indicating improved closure kinetics (**Fig. 7A**). No statistical difference was observed for the remaining experimental groups against the control (*P*>0.05, *n* = 6). To ascertain that the significant area reduction observed in wounds treated with a co-culture of both cell types in fibrin gel was due to the presence of the two different cell types, rather than to cell number, some wounds were treated with 1.35×10^5^ CD34^+^ cells encapsulated in fibrin gels. No statistically significant wound area reduction against the control was observed for this experimental condition (**Fig. S6**). At day 10, although not statistically different, wounds treated with the gel containing the co-culture of cells showed a greater area reduction compared to wounds treated with PBS. Such differences between the experimental group and control group were not observed for the remaining groups. At this stage, the wound healing in all experimental groups was characterized by reepithelialization, dermal cellular infiltration and formation of granulation tissue (**Fig. S7**).

We next evaluated the survival of the transplanted cells using a non-invasive real time fluorescence imaging (CellVizio). At day 3, cell survival was low (8.9±11.7%, *n* = 4) in wounds treated with a co-culture of CD34^+^ and CD34^+^-derived ECs. At day 10, qRT-PCR analyses of excised wounds showed the absence of human cells (data not shown). This suggests that the therapeutic effect of the cell co-culture is likely produced at the early phases of the healing process and therefore the therapeutic benefit of stem cells was not examined for a longer period of time.

### Co-transplantation of CD34^+^ cells with CD34^+^-derived ECs increases early neovascularization and decreases inflammatory processes

Because stem cells and progenies can contribute for the regeneration of the tissues through neovascularization, we assessed the capillary density by immunofluorescence using vWF as a vascular marker, at days 3 and 10 (**Figs. 7B and 7C**). At day 3, a significant increase in the density of microvessels was observed for the experimental groups containing stem cells relatively to the group of the gel alone (*P*<0.001). This indicates that part of the regenerative effect might be mediated through a neovascularization process. Although the microvessel density increased in all experimental groups at day 10, the difference between those of cell-treated wounds and controls attenuated with time (from 3 to 10 days), in line with observations from other studies of wound regeneration with stem cells [7].

The regenerative effect of stem cells and their progenies might be also mediated by a reduction of inflammation at the wound site. Therefore, the expression of inflammatory markers was assessed by qRT-PCR at day 3 post-wounding. Myeloperoxidase (MPO), CD3ε and tumor necrosis factor-alpha (TNF-α) are markers for the presence of neutrophils, T cells and inflammatory cytokines, respectively. Although not statistically significant (*P*>0.05, *n* = 9), the inflammatory reaction (considering all three markers) in the wounds treated with gel containing a co-culture of CD34^+^ cells and CD34^+^-derived ECs (**Fig. S8**) was reduced compared to the control, i.e. wounds treated with the gel alone. This effect was further confirmed at protein level, by a multiplex assay (**Fig. 7D**). In line with previous studies [16], IL-1β, TNF-α, IL-6 and GM-CSF are expressed in all experimental groups at early stages during the healing process. However, the secretion of TNF-α was statistically (*P*<0.05) higher in wounds with gel alone than in wounds treated with gel encapsulating CD34^+^ and CD34^+^-derived ECs or CD34^+^ cells alone (**Fig. 7D**). These results indicate that stem cells and their progenies are able to attenuate the inflammatory process at the site of injury, contributing to the healing progression.

## Discussion

This work reports a methodology to improve the survival, vascular differentiation and regenerative potential of UCB-derived stem cells. The methodology is based on three components that can be isolated or derived from UCB: hematopoietic stem cells (CD34^+^ cells), CD34^+^-derived ECs to improve stem cell survival and coach their differentiation into vascular cells, and fibrin gel to retain both cells at the implant site. We show that diabetic chronic wounds treated with a combination of both types of cells encapsulated in a fibrin gel have improved healing compared to wounds treated with gel containing only stem cells.

Our results indicate that fibrin gels support higher CD34^+^ cell adhesion than collagen gels, fibronectin or tissue culture polystyrene. Fibrin gels obtained by the crosslinking of fibrinogen by thrombin, two plasma-derived components [17], have RGD and AGDV sites through which they can interact with cell integrins. Our findings agree with previous studies showing that fibrin-containing thrombi provide local cues for initial adhesion of peripheral blood-derived CD34^+^ cells [18].

ECs differentiated from UCB-derived CD34^+^ cells have superior angiogenic properties than primary ECs (HUVECs). This is confirmed by their ability to secrete higher levels of angiogenic factors, including VEGF, PlGF and bFGF, and to produce a higher number of sprouts and larger network in Matrigel (**Fig. 2**). This seems to be specific for ECs derived from cord blood, since peripheral blood-derived human endothelial progenitor cells have angiogenic properties similar to HUVECs [19].

When both CD34^+^ cells and CD34^+^-derived ECs are encapsulated in fibrin gels, they crosstalk, as confirmed by the secretion of specific cytokines. CD34^+^-derived ECs encapsulated alone in fibrin gels secreted high levels of IL-6, IL-8, IL-13 and MCAF, while CD34^+^ cells encapsulated alone in fibrin gels produced high levels of IL-8, IL-13 and MCAF. The secretion profile of CD34^+^ cells is consistent with previous studies [20], [21]. When both types of cells were concurrently encapsulated in fibrin gels, they secreted two novel cytokines, cytokine IL-17 and IL-10, in addition to the ones secreted by each type of cell. IL-17 is a cytokine that has been reported to be produced by T lymphocytes and monocytes [22] and thus it is likely secreted by CD34^+^ cells, acting subsequently in ECs [23]. This is because human vascular ECs, but not CD34^+^ cells, express the IL-17 receptor (IL-17 R) [24]. The binding of IL-17 in ECs stimulates the production of IL-6, IL-8, transforming growth factor β (TGF-β) and MCAF and stimulates EC migration and tube formation [25]. IL-10 is a cytokine produced by various cell populations, including CD34^+^ cells and ECs [26]. IL-10 activity is mediated by its interaction with the IL-10 receptor, expressed on a variety of cells, including CD34^+^ cells and ECs [27]. In our study, it is unclear whether IL-10 was secreted by CD34^+^ cells or CD34^+^-derived ECs and whether IL-10 is acting on either both cells or only one type of cells. Importantly, it has been reported that IL-10 increases Bcl-2 expression and survival in CD34^+^ cells [28].

In this study, we show that the adhesion, viability, proliferation and vascular differentiation of CD34^+^ cells in fibrin gels are improved by co-culturing these cells with CD34^+^-derived ECs. Importantly, the pro-survival effect is specific for CD34^+^-derived ECs, since primary ECs (HUVECs) have no such effect. Our results also indicate that CD34^+^ cell survival, proliferation and adhesion to fibrin gels are mediated by biomolecules secreted by CD34^+^-derived ECs, since conditioned medium collected from these cells has similar inductive effects. However, CD34^+^ cell differentiation into ECs requires additional factors to the ones secreted by ECs, since CD34^+^-derived EC-conditioned medium alone does not improve CD34^+^ cell differentiation.

The improved adhesion and viability of CD34^+^ cells in the presence of CD34^+^-derived ECs is likely mediated by growth factors and cytokines. VEGF [29], IL-3 [30], IGF-I [31] and IL-10 [28] have been reported to be involved in the survival of CD34^+^ cells. We show that VEGF is highly expressed at gene level both in CD34^+^ cells and in CD34^+^-derived ECs encapsulated in fibrin gels (**Fig. 7**), and thus might account for the improved survival of CD34^+^ cells. Another factor that might contribute to the survival of CD34^+^ cells is IL-10 (see above). Our study also indicates that the improved adhesion of CD34^+^ cells in the presence of ECs is mediated by soluble factors. It has been shown that binding of soluble P-selectin, expressed by ECs, to P-selectin glycoprotein ligand-1 (PSGL-1), expressed by CD34^+^ cells, activates cellular adhesion molecules on CD34^+^ cells, particularly αvβ5 integrins [18]. The expression of these integrins favors the adhesion to fibrinogen [18]. A similar mechanism might explain the improved cellular adhesion in our co-culture system.

Our *in vitro* results show that the ERK, but not the Akt pathway, is activated in starved CD34^+^ cells treated with EC-conditioned medium. Therefore, a likely mechanism for CD34^+^ cell protection mediated by ECs is via activation of ERK. This finding is in line with recent data showing that transient activation of the ERK signaling pathway results in the survival and expansion of CD34^+^ cells [32].

CD34^+^-derived ECs, but not medium conditioned by these cells, rapidly induce the differentiation of CD34^+^ cells into ECs. CD34^+^ cells cultured for 7 days in the presence of CD34^+^-derived ECs are able to uptake DiI-labeled Ac-LDL (70% of the cells), a functional feature of ECs. These results suggest an early involvement of cell-cell interactions or extracellular matrix synthesized by ECs in the induction of vascular differentiation. Yet, the induction mechanism is still unknown. Interestingly, induction of vascular differentiation by extracellular matrix produced by ECs has been described recently for mesenchymal stem cells [33].

Diabetic ulcers are difficult to heal and innovative treatments to enhance wound healing and regeneration are needed [4]. One of the treatments being pursued involves the application of stem cells, relying on the assumption that topical application of stem cells modulates the healing response. Few studies have demonstrated the therapeutic benefit of stem cells in diabetic ulcers [7], [8]. Bone marrow-derived mesenchymal stem cells have been reported to enhance wound healing in diabetic mice, associated with increased angiogenesis [8]. This effect is mediated by factors secreted by these cells, including epidermal growth factor, keratinocyte growth factor, VEGF, bFGF, IL-6 and MCP-1 [34]. A similar paracrine effect has been documented recently for fetal progenitor cells (CD133^+^ cells) administered topically to diabetic wounds [7]. It was shown that stem cell released factors (e.g. VEGF and IL-8) stimulate angiogenesis and activate Wnt signaling, promoting the healing of diabetic ischemic ulcers.

The therapeutic effect of CD34^+^ cells or the combination of CD34^+^ cells with CD34^+^-derived ECs was tested in streptozotocin-induced diabetic mice which have previously been used to study the effect of allogeneic cell-based therapies on diabetic wound healing without immunosuppressant [7]. Although C57BL/6 mice are not immunocompromised, studies have demonstrated that human umbilical cord cells may be administered to other species for therapeutic benefit, in the absence of immune suppression, with low cross-species reactivity [35], [36]. In one case, cells have survived for 1 month after allogeneic transplantation [10] likely due to their immunological immaturity.

Importantly, no study to date has documented the regenerative potential of UCB-derived CD34^+^ cells in the context of diabetic wounds. Here, we show that the transplantation of CD34^+^ cells together with CD34^+^-derived ECs, but not CD34^+^ cells or CD34^+^-derived ECs alone, in fibrin gels enhances wound healing, compared to wounds covered with gel alone or PBS. A substantial fraction of cells (more than 90%) died over a 3-day period. This is in line with other studies showing a dramatic decline of stem cells at the injury site [7], [37]. Even so, a therapeutic benefit was observed over the 10 day period in our study, likely due to factors secreted by both cells, which increased neovascularization and decreased the inflammatory reaction. As noted before, cytokines IL-10 and IL-17 are only secreted when both cells are co-cultured. IL-10 is a potent immunosuppressant of monocyte/macrophage functions, inhibiting the production of pro-inflammatory cytokines [38]. We attempted to quantify human IL-10, IL-17 and other cytokines (IL-6, IL-8, MCF, among others) in the biopsies of wounds treated with the co-culture system for 3 days; however, the levels of all human cytokines were below the sensitivity of the Multiplex assay. The dilution effect of the human cytokines by the mouse proteins as well as the low survival of the cells at day 3 may explain the low levels of cytokines. Finally, our Multiplex results also indicate that the level of TNF-α, a pro-inflammatory cytokine, was lower for wounds treated with CD34^+^ cells and ECs, or only CD34^+^ cells encapsulated in fibrin gel.

Collectively, our data show that the therapeutic potential of hematopoietic stem cells derived from cord blood is enhanced when they are co-cultured with their vascular progenies. The crosstalk between hematopoietic stem cells and vascular cells is beneficial for their adhesion, survival, differentiation and therapeutic effect. To improve the therapeutic benefit of the cell co-culture, multiple applications, rather than a single application of the cells encapsulated in fibrin gel, could be performed. Further studies are needed to address the potential of this approach. We postulate that this co-culture system may have potential therapeutic applications, not only in chronic wounds, but also in other vascular and ischemic problems.

## Supporting Information

Figure S1 **Characterization of HUVECs by immunofluorescence.** Cells express high levels of CD31, vWF and VE-CAD, low levels of CD34, have the ability to uptake Ac-LDL and do not express typical smooth muscle cell markers such as α-SMA and SM-MHC. Bar corresponds to 20 µm.
(TIFF)

Figure S2 **Ability of vascular cells to form networks in Matrigel.** CD34^+^-derived ECs as well as HUVECs form cords when seeded in Matrigel for 48 h. Bar corresponds to 40 µm.
(TIFF)

Figure S3 **Effect of cell number on the gel contraction and in the viability of CD34^+^ cells encapsulated in fibrin gels.** A) Variation of gel diameter over time for constructs having a defined number of CD34^+^ cells. For all experimental groups, low gel contraction is observed after 10 days. B) Viability of encapsulated CD34^+^ cells, as assessed by a LIVE/DEAD assay. The assay was performed at days 2, 6 and 10. Cell viability is affected by the number of cells encapsulated in the gel. Results are average ± SD, *n* = 3 (3 readings per construct).
(TIFF)

Figure S4 **Degradation of fibrin gels containing cells.** A) Degradation of fluorescently-labeled fibrin gels containing 1×10^5^ CD34^+^ cells, or 0.35×10^5^ CD34^+^-derived ECs, or a co-culture of both cells at these numbers. Low degradation of fibrin gels is observed for all experimental groups. B) Variation of gel diameter over time. Low gel contraction is observed after 10 days. Results are average ± SD, *n* = 3. * denotes statistical significance within time group: ** *P*<0.01. # denotes statistical significance between time groups comparing the respective control/treatment groups: # *P*<0.05, ## *P*<0.01.
(TIFF)

Figure S5 **Adhesion of CD34^+^ cells to fibrin gels.** CD34^+^ cells were seeded on 24-well plates coated with fibrin gels either with (C) or without (A, B and D) CD34^+^-derived ECs (35,000 cells). The number of CD34^+^ cells per well was 100,000, except in B (135,000). The culture medium was EGM-2, except in D (EC-conditioned EGM-2). After 7 days, the cells were washed and the attached ones were counted in random microscope fields (×200 magnification). Results are average ± SD. * and *** denote statistical significance (*P*<0.05 and *P*<0.0001, respectively).
(TIFF)

Figure S6 **Regenerative effect of CD34^+^ cells encapsulated in fibrin gels on diabetic wounds.** Wound closure (relatively to initial wound area) in diabetic mice treated by topical application of 1.35×10^5^ CD34^+^ cells encapsulated in fibrin gels. Control wounds received a saline solution (PBS) only. Results are average ± SEM, *n* = 5.
(TIFF)

Figure S7 **Histological analysis of wounds treated by topical application of fibrin gel alone or fibrin gel containing CD34^+^ cells or CD34^+^ cells plus CD34^+^-derived ECs.** Representative bright-field photographs of mouse wounds at day 10, stained with hematoxylin/eosin. Bar corresponds to 100 µm.
(TIFF)

Figure S8 **Expression of inflammation-related genes by quantitative RT-PCR, on mouse wound skin biopsies at day 3.** Wounds had been treated by topical application of fibrin gel containing 1×10^5^ CD34^+^ cells and 0.35×10^5^ CD34^+^-derived ECs. Control wounds were covered with gel only. Results are average ± SD, *n* = 9.
(TIFF)

Table S1 Primer sequences used for qPCR.
(TIFF)

Materials and Methods S1 Expanded materials and methods section.
(DOC)
