# Supplementary figures and images for: Improved Survival, Vascular Differentiation and Wound Healing Potential of Stem Cells Co-Cultured with Endothelial Cells
Source: PLoS One. 2011 Jan 24;6(1):e16114. doi: 10.1371/journal.pone.0016114 (PMC3026015; doi:10.1371/journal.pone.0016114)

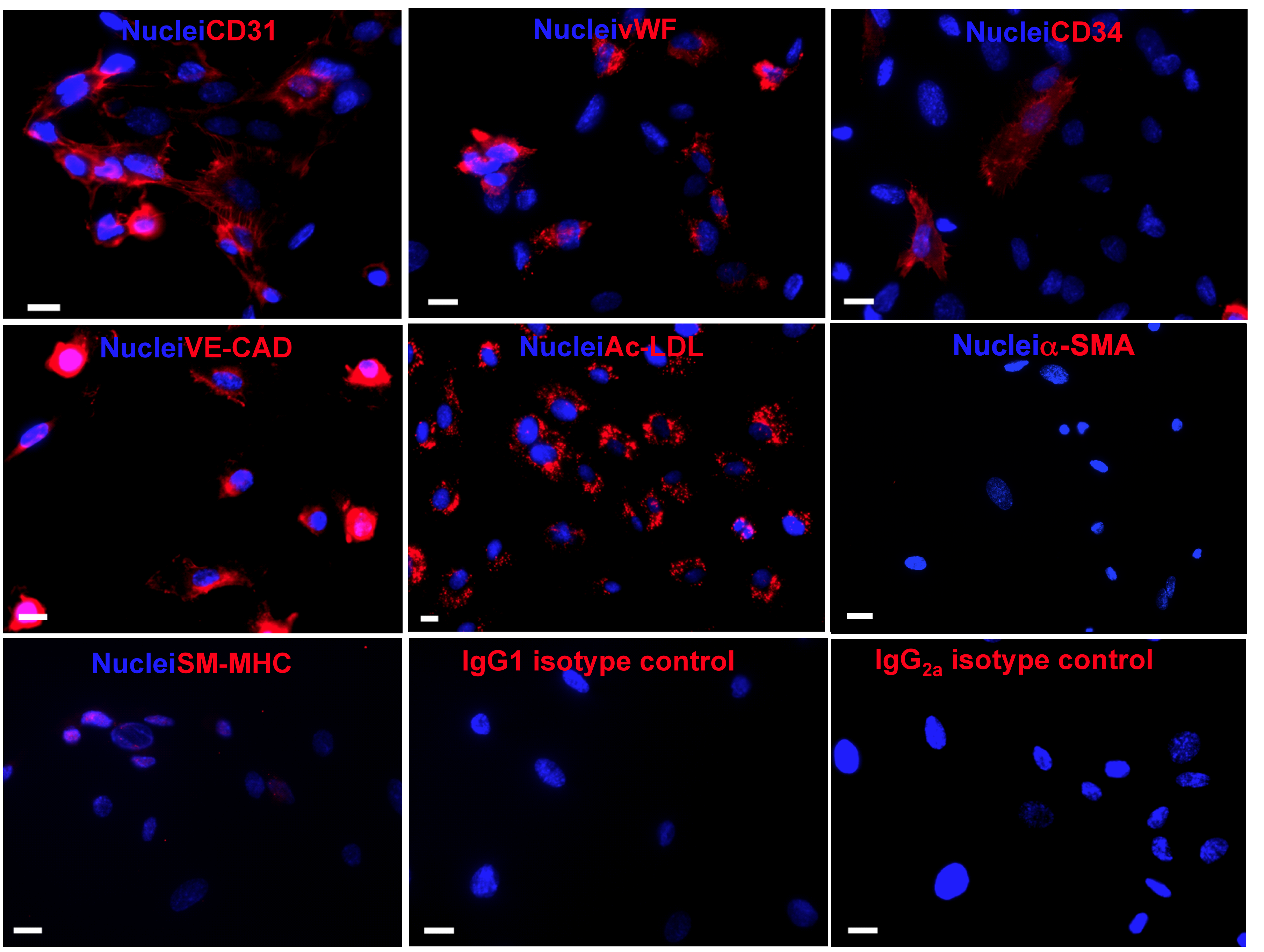

Supplement: Figure S1 — Characterization of HUVECs by immunofluorescence. Cells express high levels of CD31, vWF and VE-CAD, low levels of CD34, have the ability to uptake Ac-LDL and do not express typical smooth muscle cell markers such as α-SMA and SM-MHC. Bar corresponds to 20 µm. (TIFF) [file pone.0016114.s001.tiff]

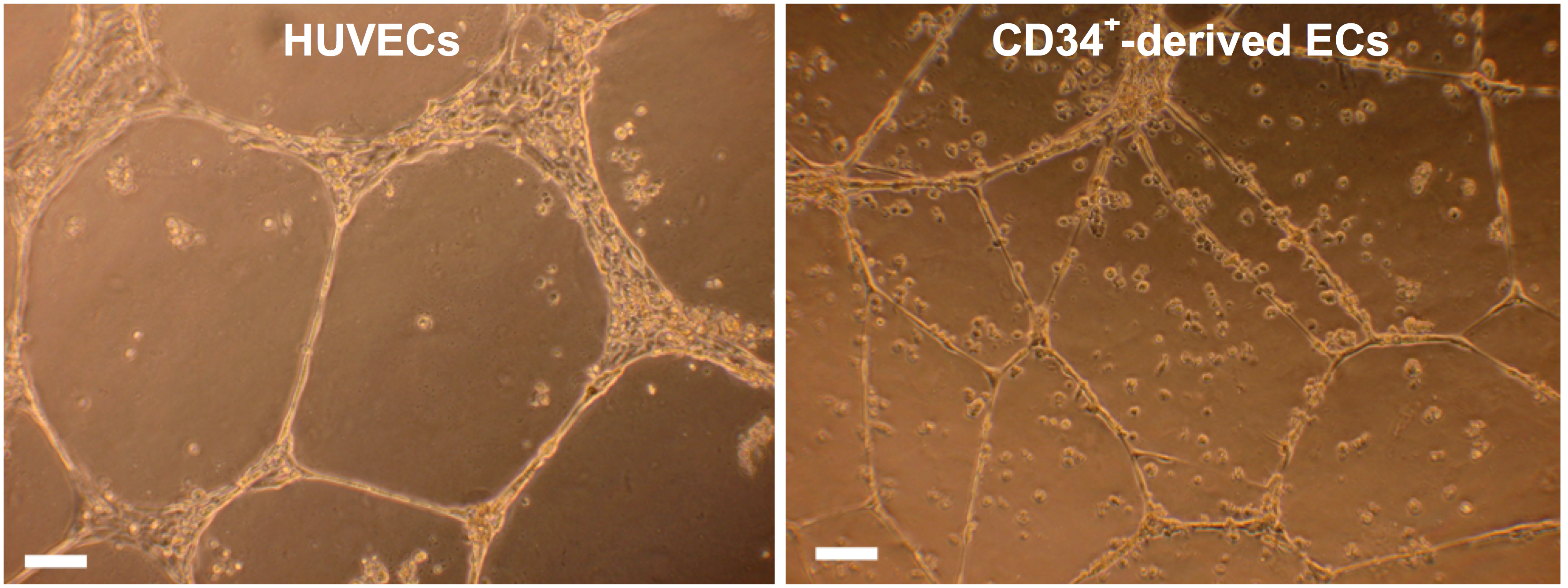

Supplement: Figure S2 — Ability of vascular cells to form networks in Matrigel. CD34+-derived ECs as well as HUVECs form cords when seeded in Matrigel for 48 h. Bar corresponds to 40 µm. (TIFF) [file pone.0016114.s002.tiff]

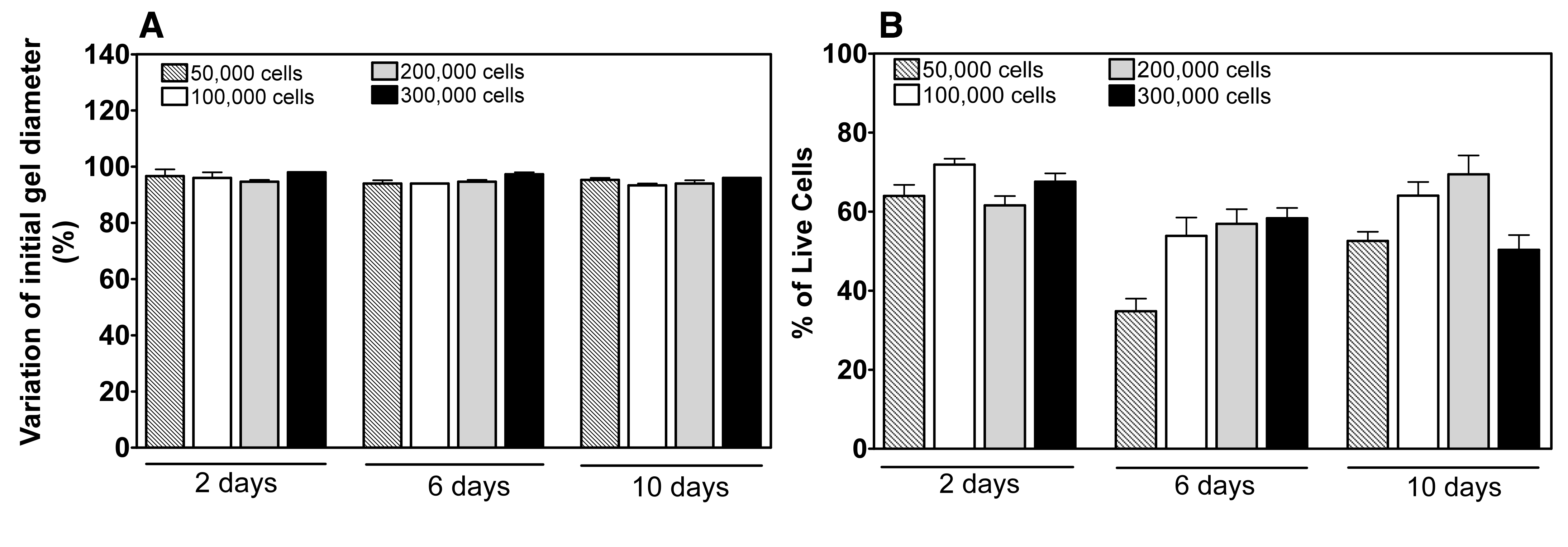

Supplement: Figure S3 — Effect of cell number on the gel contraction and in the viability of CD34+ cells encapsulated in fibrin gels. A) Variation of gel diameter over time for constructs having a defined number of CD34+ cells. For all experimental groups, low gel contraction is observed after 10 days. B) Viability of encapsulated CD34+ cells, as assessed by a LIVE/DEAD assay. The assay was performed at days 2, 6 and 10. Cell viability is affected by the number of cells encapsulated in the gel. Results are average ± SD, n = 3 (3 readings per construct). (TIFF) [file pone.0016114.s003.tiff]

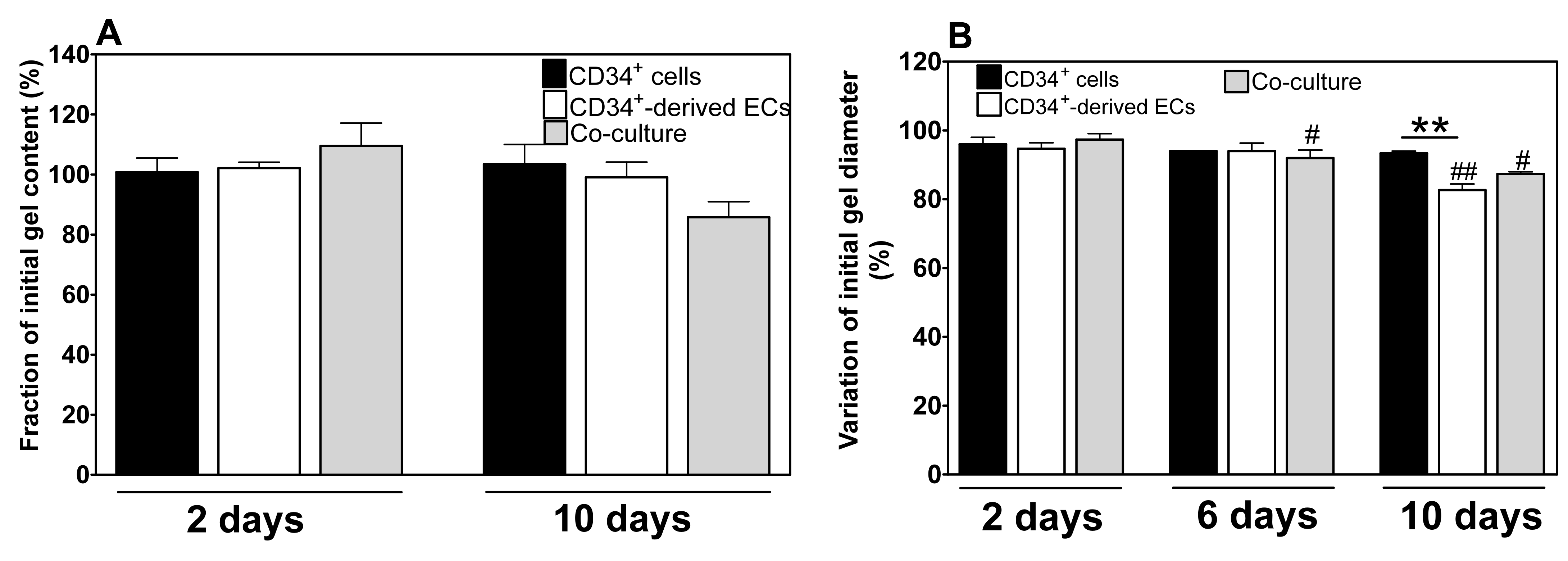

Supplement: Figure S4 — Degradation of fibrin gels containing cells. A) Degradation of fluorescently-labeled fibrin gels containing 1×10^5^ CD34+ cells, or 0.35×10^5^ CD34+-derived ECs, or a co-culture of both cells at these numbers. Low degradation of fibrin gels is observed for all experimental groups. B) Variation of gel diameter over time. Low gel contraction is observed after 10 days. Results are average ± SD, n = 3. * denotes statistical significance within time group: ** P<0.01. # denotes statistical significance between time groups comparing the respective control/treatment groups: # P<0.05, ## P<0.01. (TIFF) [file pone.0016114.s004.tiff]

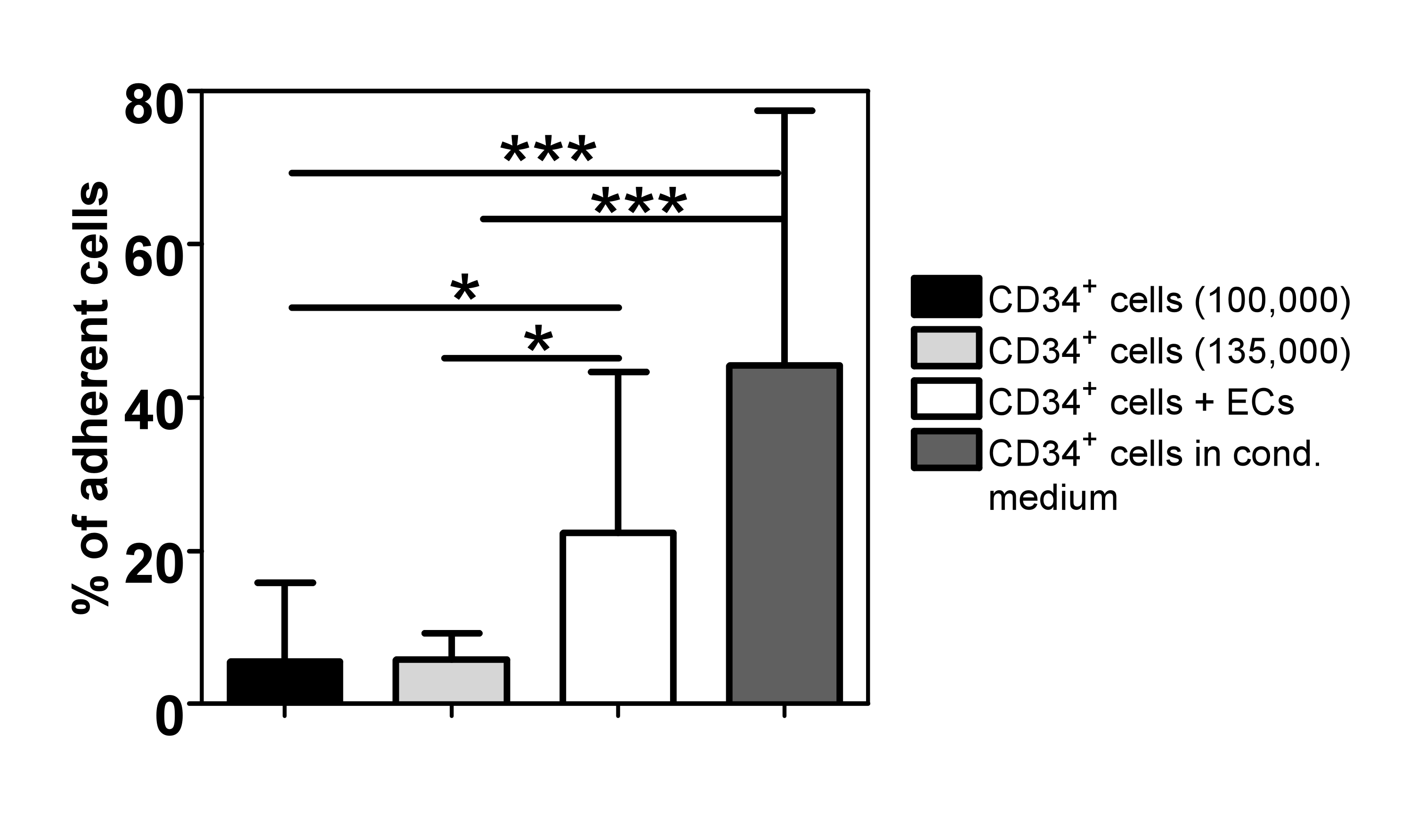

Supplement: Figure S5 — Adhesion of CD34+ cells to fibrin gels. CD34+ cells were seeded on 24-well plates coated with fibrin gels either with (C) or without (A, B and D) CD34+-derived ECs (35,000 cells). The number of CD34+ cells per well was 100,000, except in B (135,000). The culture medium was EGM-2, except in D (EC-conditioned EGM-2). After 7 days, the cells were washed and the attached ones were counted in random microscope fields (×200 magnification). Results are average ± SD. * and *** denote statistical significance (P<0.05 and P<0.0001, respectively). (TIFF) [file pone.0016114.s005.tiff]

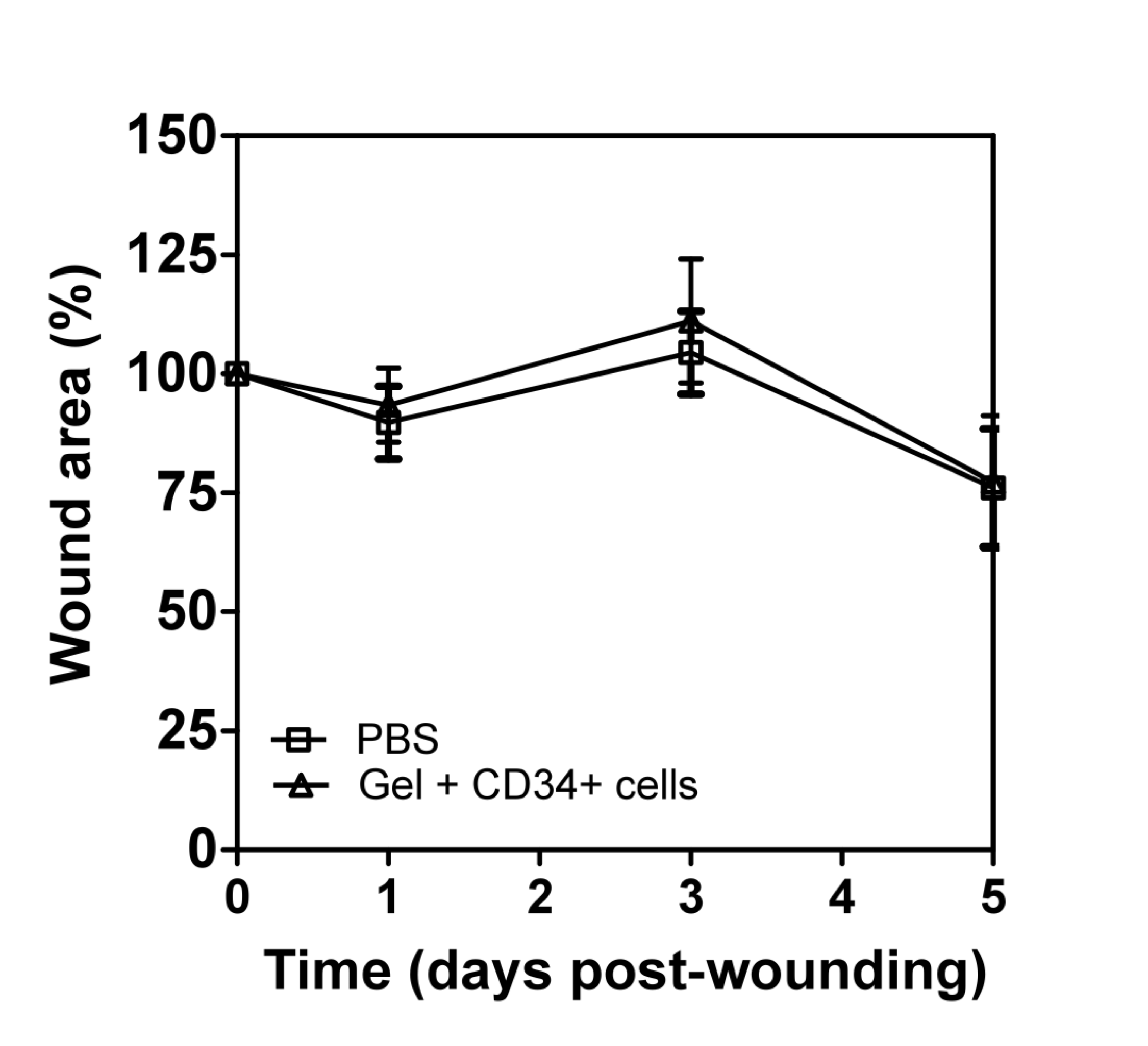

Supplement: Figure S6 — Regenerative effect of CD34+ cells encapsulated in fibrin gels on diabetic wounds. Wound closure (relatively to initial wound area) in diabetic mice treated by topical application of 1.35×10^5^ CD34+ cells encapsulated in fibrin gels. Control wounds received a saline solution (PBS) only. Results are average ± SEM, n = 5. (TIFF) [file pone.0016114.s006.tiff]

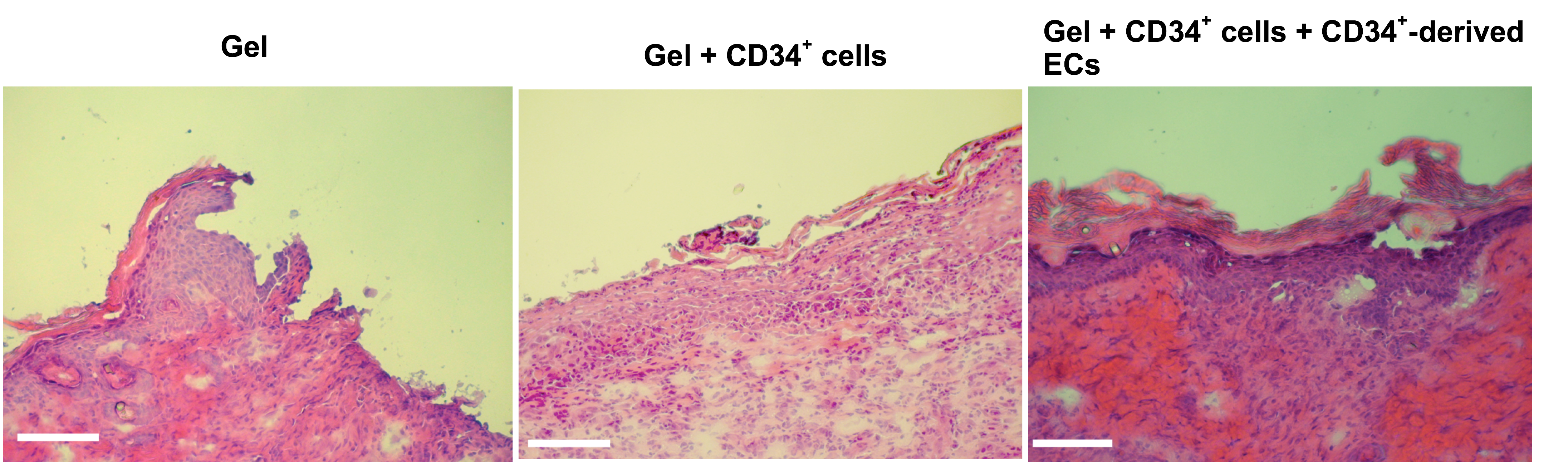

Supplement: Figure S7 — Histological analysis of wounds treated by topical application of fibrin gel alone or fibrin gel containing CD34+ cells or CD34+ cells plus CD34+-derived ECs. Representative bright-field photographs of mouse wounds at day 10, stained with hematoxylin/eosin. Bar corresponds to 100 µm. (TIFF) [file pone.0016114.s007.tiff]

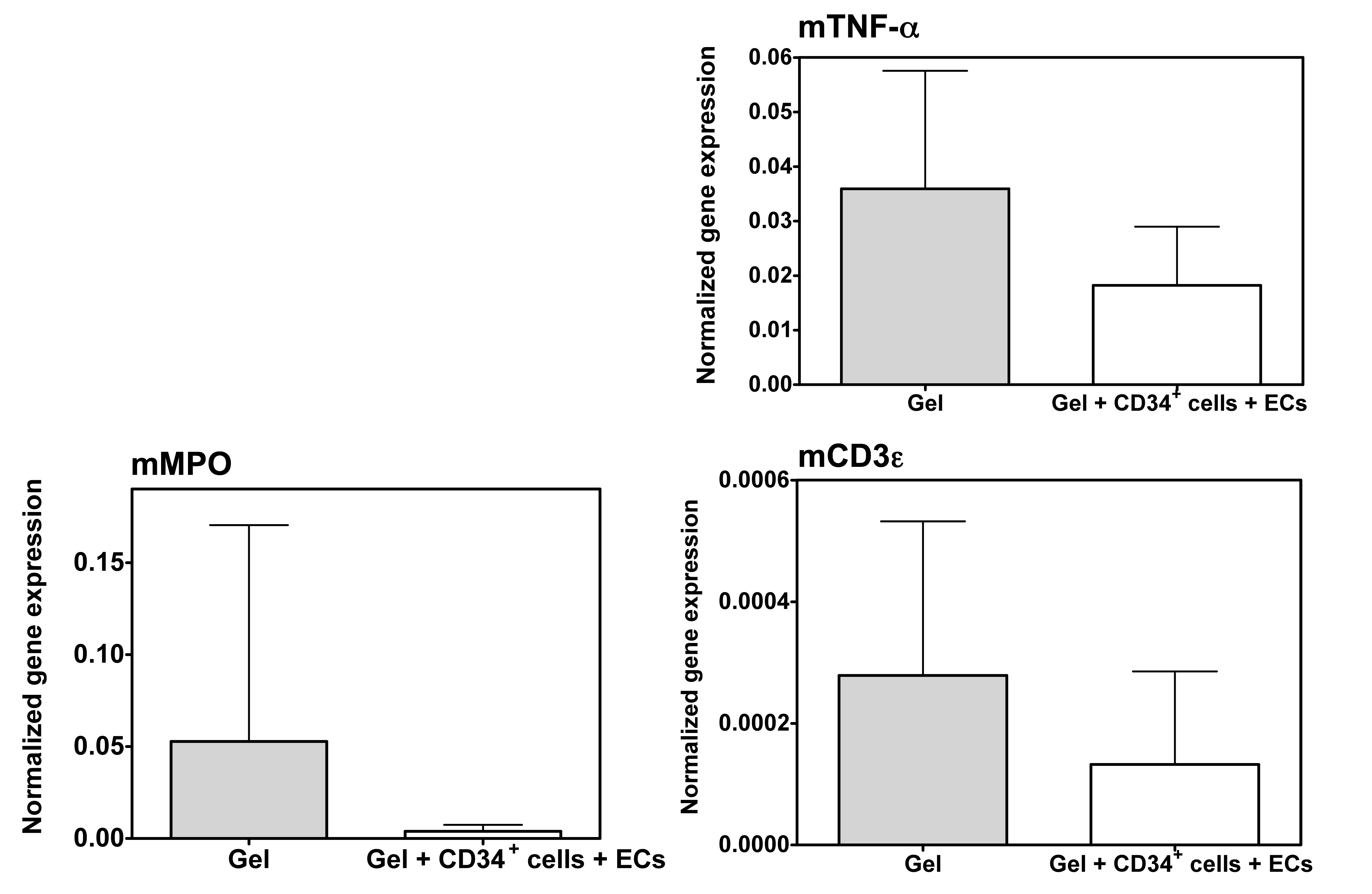

Supplement: Figure S8 — Expression of inflammation-related genes by quantitative RT-PCR, on mouse wound skin biopsies at day 3. Wounds had been treated by topical application of fibrin gel containing 1×10^5^ CD34+ cells and 0.35×10^5^ CD34+-derived ECs. Control wounds were covered with gel only. Results are average ± SD, n = 9. (TIFF) [file pone.0016114.s008.tiff]

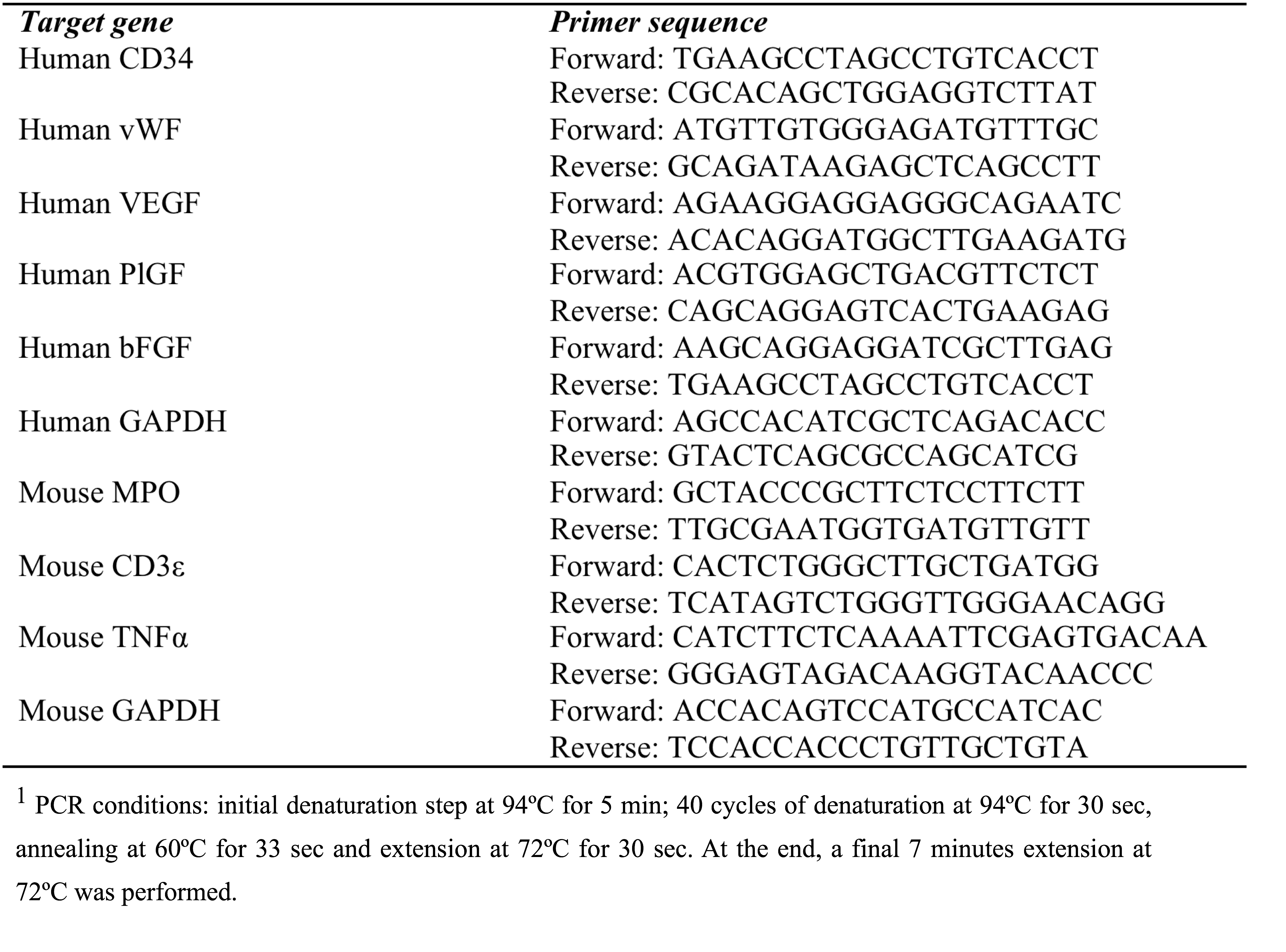

Supplement: Table S1 — Primer sequences used for qPCR. (TIFF) [file pone.0016114.s009.tiff]
