# Supplementary material for: Improved Survival, Vascular Differentiation and Wound Healing Potential of Stem Cells Co-Cultured with Endothelial Cells
Source: PLoS One. 2011 Jan 24;6(1):e16114. doi: 10.1371/journal.pone.0016114 (PMC3026015; doi:10.1371/journal.pone.0016114)
Supplement: Materials and Methods S1 — Expanded materials and methods section. (DOC) [file pone.0016114.s010.doc]

**MATERIALS and METHODS S1**

**Staining of live cells with fluorescent dyes**

Cells were ressuspended in serum-free medium (M199) at a density of 1 × 10^6^ cells/mL and 5-10 μL of the dye [5(6)-carboxyfluorescein diacetate N-succinimidyl ester (CFSE; Sigma-Aldrich) or Vybrant® DiD cell-labeling solution (Invitrogen)] was added per mL of cell suspension, mixing well by gentle pipetting. After incubation at 37ºC for up to 30 minutes, the labeled cell suspension was centrifuged, the supernatant removed and the cells ressuspended in warm (37ºC) M199 medium. The cells were then washed twice in warm medium and finally ressuspended in the culture medium of choice. For cells adherent to culture plates or flasks, a similar procedure was followed, with the required adjustments. CFSE forms green fluorescent conjugates on deacetylation, whilst DiD-labeled cells display red fluorescence.

**Matrigel assay**

A 24-well plate was coated with 0.4 mL of Matrigel (BD Biosciences) per well and incubated for 30 minutes at 37ºC. Either CD34^+^-derived endothelial cells (passage 4) or HUVECs were seeded on top of the polymerized Matrigel at a concentration of 1 × 10^5^ per 300 μL of EGM-2 medium. The cells were either unlabeled or previously labeled with CFSE or Vybrant® DiD. After 1 h of incubation at 37ºC, 1 mL of EGM-2 was added. Cord formation was evaluated by phase contrast microscopy (Zeiss Axiovert 40C, Carl Zeiss International, Germany, www.zeiss.com), 15 or 48 h after cell seeding.

**Preparation of fibrin gels**

Fibrin gels were formed by crosslinking of fibrinogen in the presence of thrombin (both from Sigma-Aldrich). The fibrinogen solution was prepared by dissolving human fibrinogen in Tris-buffered saline (TBS) (Sigma-Aldrich), pH 7.4 (20 mg/mL), and then sterilized by filtering through a 0.22 μm syringe filter (Acrodisc, Pall, NY, USA). Fresh thrombin solutions were prepared by dissolving human thrombin in TBS at pH 7.4 at a concentration of 50 U/mL. Fibrin gels (50 μL, unless otherwise stated) were prepared by mixing three different components: fibrinogen (10 mg/mL), CaCl_2_ (Merck, NJ, USA) (2.5 mM) and thrombin (2 U/mL). This solution was allowed to gel at 37ºC and 100% relative humidity.

**Degradation of fibrin gels**

Gel precursor solution was prepared by mixing Alexa Fluor® 488 human fibrinogen conjugate (Invitrogen) (0.156 mg) to unlabeled fibrinogen (9.844 mg) in 1 mL of TBS. The degradation rate of fibrin gels with or without cells over time was indirectly estimated by the decrease of their fluorescence. Their fluorescence was measured immediately at time zero and at the desired time points. Complete degradation of the gels was induced by incubation with 200 μL of a solution of human plasmin (Sigma-Aldrich) in TBS (0.006 U per gel) for an overnight at 37ºC. Following centrifugation, the fluorescence of the supernatant fractions was measured at 520 nm in a SPECTRAmax Gemini EM fluorescence microplate reader (Molecular Devices, Sunnyvale, CA, USA, [www.moleculardevices.com](http://www.moleculardevices.com/)).

**Viability and metabolic activity of cell constructs**

Cell viability of cell constructs was determined using a LIVE/DEAD kit (Invitrogen). The gels containing the encapsulated cells were washed in PBS, immersed in a working solution of 2 μg/mL calcein AM and 4 μg/mL ethidium homodimer-1 in PBS for 30 minutes at 37ºC and visualized under a Zeiss Axiovert 200M fluorescence inverted microscope.

The metabolic activity of cell constructs was measured through a 3-(4,5-dimethylthiazol-2-yl)-2,5-diphenyl tetrazolium bromide (MTT, Sigma-Aldrich) assay after 2, 6 and 10 days of culture. The MTT solution (1 mL, 0.5 mg/mL in the same type of medium in which the cells had been cultured) was added to each well containing the cell constructs and incubated for 4 h, at 37ºC. After that time, the cell constructs were placed in 1.5 mL polypropylene tubes and 0.15 mL of dymethyl sulfoxide (DMSO; Merck) was added. The constructs were broken apart using a tissue homogeneizer, to release the formazan crystals formed by cells presenting mitochondrial metabolic activity. The formazan crystals were solubilized in the DMSO and their absorbance measured spectrophotometrically at 540 nm, in a microplate spectrophotometer (PowerWave XS, BioTek, Winooski, VT, USA, www.biotek.com).

**FACS analysis**

Cells were dissociated from the culture plate by exposure to Cell Dissociation Buffer (Invitrogen) for 5-10 minutes and gentle pipetting, centrifuged and finally ressuspended in PBS supplemented with 5% (v/v) FBS. The single cell suspensions were aliquoted (1.25-2.5 × 10^5^ cells per condition) and stained with either isotype controls or antigen-specific antibodies: anti-human PECAM1-FITC (BD Biosciences Pharmingen), CD14-FITC, CD34-PE, CD45-FITC (all from Miltenyi Biotec) and KDR/Flk1-PE (R&D Systems, Minneapolis, USA). Cells were analyzed without fixation on a FC500 flow cytometer (Coulter, USA, www.beckmancoulter.com), using propidium iodide (7-AAD Viability staining solution; bioNova científica, Madrid, Spain) to exclude dead cells. Data analysis was carried out using Coulter FC Analysis software.

**Immunostaining**

Cells were fixed with 4% (v/v) paraformaldehyde (EMS, Hatfield, USA) for 15-20 minutes at room temperature. After permeabilizing the cells with 0,1% (v/v) Triton X-100 (Sigma-Aldrich) for 10 minutes, whenever required, and blocking for 30 minutes with 1% (w/v) bovine serum albumin (BSA) solution (Sigma-Aldrich), the cells were stained for 1 h with the following primary mouse anti-human monoclonal antibodies: PECAM1, CD34, von Willebrand factor (vWF), α-smooth muscle actin (α-SMA), smooth muscle myosin heavy chain (SM-MHC) (all from Dako, Glostrup, Denmark) and VE-cadherin (VE-CAD) (Santa Cruz Biotechnology, Santa Cruz, USA). In each immunofluorescence experiment, an isotype-matched IgG control was used. The binding of primary antibodies to specific cells was detected with anti-mouse IgG Cy3 conjugate (Sigma-Aldrich). The nucleus of cells was stained with 4',6-diamidino-2-phenylindole (DAPI; Sigma-Aldrich). After the indirect labelling, the cells were examined with a Zeiss fluorescence microscope.

For uptake of DiI-labeled acetylated low-density lipoprotein (DiI-Ac-LDL), cells were incubated with 10 μg/mL DiI-labeled Ac-LDL for 4 h at 37ºC. After incubation, cells were washed three times in EGM-2, fixed with 4% (v/v) paraformaldehyde for 30 min and visualized in a fluorescence microscope.

**Quantitative reverse transcription-polymerase chain reaction (qRT-PCR) analysis**

Cell constructs were frozen and ground to a fine powder in a cold mortar, under liquid N_2_, transferred to polypropylene tubes, homogenyzed in Trizol reagent (Invitrogen) and total RNA was extracted by using the RNeasy Mini Kit (Qiagen, Valencia, USA), according to manufacturer’s instructions. When the starting material was frozen mouse skin, tissue samples were disrupted in Trizol in 2 mL tubes containing a 5 mm diameter stainless steel bead (Qiagen), in a TissueLyser II aparatus (Qiagen, www1.qiagen.com) for 2 min, at 30 Hz (twice). When starting from a cell suspension, cells were centrifuged and homogenized in Trizol. In all cases, cDNA was prepared from 1 μg total RNA using Taqman Reverse transcription reagents (Applied Biosystems, Foster City, USA). Quantitative PCR (qPCR) was performed using Power SYBR Green PCR Master Mix (Applied Biosystems) and the detection was carried out in a 7500 Fast Real-Time PCR System (Applied Biosystems, www.appliedbiosystems.com). Quantification of target genes was performed relatively to the reference (human or mouse, depending on the type of cells under analysis) GAPDH gene: relative expression = 2^[-(Ct^_sample_^-Ct^_GADPH_)]. The mean minimal cycle threshold values (Ct) were calculated from four independent reactions. Primer sequences are published as supporting information (**Table S1**).

**Cytokine secretion analyses**

Cell culture supernatants and protein isolates were evaluated for the presence and concentrations of cytokines using either a Bio-Plex Pro Human Cytokine 17-Plex Panel Assay or a Bio-Plex Pro Mouse Cytokine 8-Plex Assay (both from Bio-Rad, Hercules, CA, USA), according to manufacturer’s instructions, in a Bio-Plex 200 System (Bio-Rad, www.bio-rad.com). The human Group I 17-Plex Panel consisted of the following analytes: interleukin-1β (IL-1β), IL-2, IL-4, IL-5, IL-6, IL-7, IL-8; IL-10, IL-12(p70), IL-13, IL-17, granulocyte colony-stimulating factor (G-CSF), granulocyte/macrophage colony-stimulating factor (GM-CSF), interferon-γ (IFN-γ), monocyte chemotactic protein (monocyte chemotactic activating factor [MCP-1 (MCAF)], macrophage inﬂammatory protein-β (MIP-1 β) and tumor necrosis factor-α (TNF-α). The mouse group I 8-Plex panel comprised the following analytes: IL-1 β, IL-2, IL-4, IL-5, IL-10, GM-CSF, IFN-γ and TNF-α. A mouse Singleplex/x-Plex IL-6 Bead Region was combined to these analytes. Supernatant media samples were collected, centrifuged to remove precipitates and frozen. Proteins were isolated from frozen mouse skin tissue samples that were disrupted in a TissueLyser II aparatus (Qiagen) for 2 min, at 30 Hz (twice), by using the Bio-Plex Cell Lysis kit (Bio-Rad), according to the manufacturer’s recommendations. A standard range of 0.2 to 3,200 pg/mL was used. Samples and controls were run in triplicate, standards and blanks in duplicate.

**Analysis of total and phosphorylated Akt and ERK protein levels**

Activation of Akt and extracellular signal-regulated kinase (ERK) in CD34^+^ cells and CD34^+^-derived ECs was promoted by starving the cells for 19 h in serum-free M199 medium, with Earle's Salts, L-glutamine (Sigma-Aldrich) and then treating them for 10 minutes, by replacing the medium by cell-conditioned M199 or fresh M199. Conditioned M199 used for CD34^+^ cell treatment had been obtained by culturing ECs in M199 for 24 h (0.75 × 10^5^ cells per mL of medium), removing the medium and sterilizing it by filtration. Conditioned M199 used for EC treatment had been obtained by culturing CD34^+^ cells in M199 for 24 h (2 × 10^5^ cells per mL of medium), removing the medium and sterilizing it by filtration. Following treatment, proteins were isolated from the cells, either adherent to plates or in suspension, using the Bio-Plex Cell Lysis kit (Bio-Rad), according to the manufacturer’s recommendations. The levels of Akt and ERK phosphorylation were determined using Bio-Plex kits from Bio-Rad, according to manufacturer’s instructions.

**Determination of apoptosis and necrosis in CD34^+^ cells**

To determine CD34^+^ apoptosis and necrosis, 0.30 × 10^5^ CD34^+^ cells were cultured on top of 500 μL fibrin gels, in 24-well plates, in either EGM-2 medium or EGM-2 conditioned by ECs. After 7 days of culture, cell apoptosis/necrosis was assessed using the Vybrant® Apoptosis Assay Kit #3 (FITC annexin V/propidium iodide (Invitrogen), according to manufacturer’s recommendations. This assay detects the externalization of phosphatidylserine in apoptotic cells. In normal live cells, phosphatidylserine is located on the cytoplasmatic surface of the cell’s membrane. Propidium iodide stains necrotic cells with red fluorescence.

**Animals**

All protocols in this study were approved by the Ethics Committee of the Faculty of Medicine of the University of Coimbra. Male C57BL/6 wild-type mice (10-12 week-old), purchased from Charles River (Wilmington, MA, USA) and weighing between 20 and 30 g, were housed in a conventional animal facility on a 12 h light/12 h dark regimen and fed a regular chow *ad libitum*.

**1- Induction of diabetes and dermal wounds**

Diabetes mellitus was induced in mice by a single intraperitoneal injection of 150 mg/kg of streptozotocin (STZ; Sigma-Aldrich), in 200 µL citrate buffer, pH 4.2, and the animals were used 6-8 weeks after induction of a diabetic metabolic state. Glycemia was weekly monitored after STZ treatment with a glucometer with glucose test strips (Accu-Chek Aviva, Roche) and only animals with blood glucose levels greater than 300 mg/dL were used in this study. Insulin (16-32 U/Kg or 0.4-0.8 U/mouse, Sigma-Aldrich) was injected only for weight maintenance.

To evaluate wound healing response to treatment with stem cells in scaffolds, mice were subjected to the creation of dermal wounds 6-8 weeks following diabetes induction. The animals were anesthetized by intramuscular injection of a xylazine /ketamine solution [ketamine hydrochloride, 10 mg/mL, (Imalgene®, Merial, Barcelona, Spain), 50mg/kg of body weight, and xylazine hydrochloride, 2 mg/mL, (Rompun®, Bayer Healthcare, Germany), 10mg/kg of body weight] and allowed to recover on a hot pad (37°C). The hair was shaved in the dorsolumbar skin and two 6 mm-diameter full-thickness excisional wounds, extending to the adipose tissue, were performed with a sterile biopsy punch, after disinfecting the area with a povidone-iodine solution (Betadine®). The wounds were longitudinally aligned and separated by a sufficient amount of non-wounded skin.

**2- Wound treatment**

The putative therapeutic effect of stem cells and their progenies encapsulated in fibrin gels on wound healing was assessed by a single topical application of the treatment of choice on the wounds immediately after they were created. In each animal, a 25 μL fibrin gel precursor containing 1 × 10^5^ human umbilical cord blood CD34^+^ cells, either alone or in combination with 0.35 × 10^5^ CD34^+^-derived endothelial cells, or 0.35 × 10^5^ CD34^+^-derived ECs, was applied on one of the wounds, in either the top (in half of the animals) or in the bottom position, and allowed to polymerize, whereas on the other wound only fibrin gel without cells (in some experimental groups) or PBS (in the remaining groups) was applied, as an internal control. All cells had been previously labelled with CFSE.

The progress of wound closure was monitored by measuring the wound area. To determine the rate of wound closure, the excision wounds were traced on a transparent paper having a millimeter scale, wound areas evaluated using computer imaging analysis (AxioVision 4.8, Zeiss) and the change in wound area was calculated as the percentage of wound area that had healed. Measurements were done immediately after wounding, as well as one, three, five, eight and 10 days later. The wounds were photographed at the same time points.

*In vivo* bio-distribution of cells at day 0 and day 3 was monitored by noninvasive fiber-based confocal microscopy (CellVizio®, Mauna Kea Technologies, Newtown, PA, USA, www.maunakeatech.com). The S-1500 optical probe used for image acquisition has a diameter of 1.5 mm, providing images immediately below the surface of biological tissue, with a slice thickness of 15 µm and a lateral resolution of 5 µm. Fluorescent-labeled cells localized in the wounds were quantified in at least 3 mice.

**3- Analyses of excision wounds**

Animals were anesthetized and sacrificed by cervical dislocation either on the third or tenth day post-wounding and 10 mm-diameter skin biopsy specimens, centered on the wound bed and comprising the wound margins, were collected.

Each biopsy sample was cut in two halves, one of which was embedded in cryomolds filled with O.C.T. (Tissue Tek®/Shandon Cryomatrix®; Thermo Fisher Scientific, Waltham, MA, USA), frozen in dry-ice and cryopreserved at -70°C, whereas the other half was snap-frozen in liquid nitrogen and also stored at -70°C. O.C.T.-embedded samples were serially sectioned in 7 μm slices in a Leica CM3050 S cryostat (Leica, Wetzlar, Germany, [www.leica-microsystems.com](http://www.leica-microsystems.com/)), cooled to -20°C, and some were immunostained for vWF to determine total capillary density (human and mouse). Staining with rabbit anti-vWF (1:300; Dako) was followed by goat Cy3-conjugated anti-rabbit IgG (1:60; Sigma-Aldrich). qPCR analysis was performed on the remaining skin biopsy samples, to determine the total number of human cells. Other sections underwent histological staining with hematoxylin and eosin (H&E).
